# Understanding the social and physical menstrual health environment of secondary schools in Uganda: A qualitative methods study

**DOI:** 10.1371/journal.pgph.0002665

**Published:** 2023-11-29

**Authors:** Andrew Sentoogo Ssemata, Denis Ndekezi, Catherine Kansiime, Robert Bakanoma, Clare Tanton, Kate Andrews Nelson, Laura Hytti, Stella Neema, Belen Torondel, Janet Seeley, Helen A. Weiss, on behalf of the MENISCUS Group

**Affiliations:** 1 MRC/UVRI and LSHTM Uganda Research Unit, Entebbe, Uganda; 2 Faculty of Public Health Policy, Department of Global Health & Development, London School of Hygiene & Tropical Medicine, London, United Kingdom; 3 Faculty of Epidemiology and Population Health, MRC International Statistics & Epidemiology Group, London School of Hygiene & Tropical Medicine, Keppel Street, London, United Kingdom; 4 Department of Sociology & Anthropology, School of Humanities and Social Sciences Makerere University, Kampala, Uganda; 5 Faculty of Infectious and Tropical Diseases, Department of Disease Control, London School of Hygiene & Tropical Medicine, Keppel Street, London, United Kingdom; Tata Institute of Social Sciences, INDIA

## Abstract

Adolescent girls face social, psychological, and physical problems managing menstruation in schools in low-resource settings. This study aimed to evaluate the social and physical menstrual health environment of secondary schools in Wakiso and Kalungu districts, Uganda, in preparation for a subsequent menstrual health intervention trial to improve education, health and wellbeing. We conducted a qualitative rapid assessment in 75 secondary schools in Uganda. This involved conducting in-depth interviews with 150 head/senior teachers and 274 students, 26 Focus Group Discussions with students, and 13 transect walks to observe school Water, Sanitation and Hygiene (WASH) facilities between May and October 2021. Due to COVID-19 related school closures, face-to-face research activities were halted and in-depth interviews were conducted over phone and replaced focus group discussions. We employed a thematic framework analysis approach using the social-ecological model (which focuses on the complex interplay between individual, interpersonal, institutional, and societal factors) to generate themes and key concepts. Participants described the social and physical menstrual health environment of secondary schools at the individual level (knowledge gaps on menstruation before menarche, negative norms and beliefs about menstrual health); interpersonal level (limited psycho-social support, myths and misconceptions about the disposal of sanitary materials and pain relief, menstrual hygiene management (MHM) support from school nurses, peers and senior teachers); institutional level (non-implementation of Government circulars on MHM, lack of school-level guidelines policies and programs on MHM and poor WASH facilities, i.e. lack of soap, safe water and unclean toilets); and societal level (MHM programmes provided by civil society groups, health workers, and students’ school associations). The findings showed individual, societal and institutional burdens related to menstrual experiences. Multi-level evidence-based interventions aimed at improving the social and physical environment for menstrual health among school-going girls are needed.

## Introduction

Menstrual health is defined as complete physical, mental, and social well-being in relation to the menstrual cycle [1]. Adolescent girls face social, psychological, and physical problems managing menstruation [1,2]. For many adolescent girls in low and middle-income countries, including Uganda, challenges associated with menstrual hygiene management (MHM) at school include lack of access to hygienic absorbents, inadequate facilities to change, lack of separate toilets with doors that can close safely, lack of access to soap and water, and lack of privacy and proper disposal of sanitary pads [1]. In addition, inadequate social support and culturally restrictive menstrual management practices can lead to internalised menstrual stigma and hence experiences of shame, distress and lack of confidence around menstruation [1]. This can impact on broader psychosocial well-being and mental health (i.e. impacts on anxiety, depression and wellbeing beyond the immediate menstrual experience) and poor self-esteem, which can in turn impact on social participation, education and employment [1–6]. Improving secondary education for girls is a global priority and a key determinant of health and development through multiple pathways, including income, early marriage and childbearing, health, and agency and decision-making [7]. The challenge of managing menstruation is one factor that can affect girls’ ability to succeed and thrive [8].

Pilot studies in Kenya, Gambia and Uganda suggest that multicomponent school-based menstrual health interventions have the potential to improve reproductive health, mental health and wellbeing, and reduce psychosocial stress and increase opportunities for accessing school, among adolescent schoolgirls [9–11]. However, there is little robust evidence on whether school interventions improve education, health and well-being [12]. A key priority of the “MHM in Ten” initiative to advance the menstrual health agenda in schools by 2024 is to generate robust evidence on whether improvement in menstrual health increases girls’ attendance and attainment at school [13–15].

There are legal and policy frameworks, programmes and activities to support MHM implementation. These range from global to national and school-level frameworks. In Uganda, the Government has committed to improving menstrual health by forming a National MHM Steering Committee, holding the first international MHM conference in 2014, launching the Menstrual Hygiene Management Charter in 2015 [15]. A number of Ugandan Government ministries and civil society organisations are committed to promoting the rights to menstrual health of girls and women through celebrating International MHM Day and conducting research on and promoting private/public sector partnership in MHM to sustain effective advocacy, resources allocation and increased access to affordable and appropriate sanitary materials. In 2015, the Ministry of Education and Sports (MoES) issued a circular to all primary and secondary schools with instructions for improving menstrual health (Circular No. 1/2015). The Circular recommends provision of clean, private, toilet facilities; regular supply of water and soap; emergency supplies of pads and painkillers; training of teachers, health assistants, and inspectors; and involvement of parents in supporting and providing menstrual health information and materials. [16]. A review conducted in Uganda in 2018 among 137 primary and secondary schools found that although 98 (94%) schools received this Circular, only 23 (17%) were implementing the guidelines [16]. Reasons for this included poor social and physical school environments, limited budgets for menstrual health provision, unclear roles and responsibilities for menstrual health within schools, and menstrual health issues being left entirely to the senior women and senior men teachers at the school level [17,18].

In 2015–2016, we conducted a mixed-methods study that showed that poor menstrual health was a key contributory factor, along with poverty, for girls missing secondary school in Wakiso district, central Uganda (MENISCUS-1) [13]. The study showed an unmet need for effective interventions to enable girls to manage better both the psychosocial aspects of menstruation (anxiety, stigma and distress) and physical aspects (pain management, water, sanitation and hygiene (WASH) environment, and use of appropriate menstrual materials) [13]. Qualitative data showed that poor knowledge of menstruation led to embarrassment and a fear of teasing. This social context, together with the lack of adequate menstrual health materials and a poor WASH environment, led to school absenteeism by girls. Following this, we co-developed a school-based menstrual health intervention with stakeholders, and piloted this in two schools in Wakiso District in 2018–2019 (MENISCUS-2) [3]. The intervention addressed health promotion (education, attitudes, well-being) and goods (menstrual products, improved WASH facilities, pain management), and was feasible to deliver and highly acceptable to stakeholders. This led to development of a cluster-randomised trial in 60 secondary schools in Wakiso and Kalungu districts to evaluate the effectiveness of the MENISCUS intervention on education, health and wellbeing outcomes [9].

Previous research on menstrual health in Uganda has not evaluated the social and the physical menstrual health environment of secondary schools [3,17]. The objectives of this study were to address this evidence gap and prepare for the cluster-randomised trial, by describing (i) the current status of menstrual health programmes being implemented in schools, (ii) the social and physical school environment, including access and type of basic WASH facilities and school facilities for general illness management as these are relevant in menstrual health management such as management of menstrual pain, and (iii) developing an understanding of the interactions between school staff, students and parents and the social and physical school environment relating to menstrual health.

### Theoretical orientation

We adopted the social-ecological model (SEM) as a theoretical framework for this study [19]. The SEM framework uses five interdependent levels—individual, interpersonal, organizational, community and public policy/structural factors to examine the influence on health behaviour at each level. This framework has been used to review the state of knowledge on menstrual health and hygiene in Nepal [20]. Recognising that menstrual health is not limited to individual behaviours, we adopted the SEM as a useful framework to critically examine the social and the physical menstrual environment in Ugandan secondary schools focusing on four levels–individual, interpersonal, institutional and societal.

## Materials and methods

### Study design and data collection methods

The qualitative rapid assessment methodology is an intensive team-based set of methods used in a particular combination to capture the main characteristics of specific communities [21–23]. In this study, we used the rapid assessment methodology to document the interaction between people (teachers and students) and place (school), to investigate the social and physical menstrual health environments within secondary schools and their communities in preparation for a subsequent menstrual health intervention trial in schools from Wakiso and Kalungu districts.

The eligibility criteria for inclusion of schools in the rapid assessment were: mixed-sex secondary schools with Secondary 1 (S1)-S4 classes; day or mixed day/boarding schools; at least minimal WASH facilities (including an improved water source and sex-specific sanitation facilities that are functional, usable, and accessible), estimated enrolment of approximately 50–125 female S1 students (Year 1 of secondary school) as of January 2020 (before the COVID-19 lockdown) in Wakiso, and 40–125 female students in S1 in Kalungu, based on the 2019 report on the Master List of Education Institutions in Uganda as the primary determinant of size eligibility [24]. Exclusion criteria included: schools that were participating in a menstrual health related programme; boarding schools with no day students; single-sex schools and schools exclusively for students with disabilities. Schools where the head teacher reported substantially smaller enrolment prior to COVID-19 lockdown closures (>30% below the eligibility cut-off) were deemed ineligible.

A stakeholder workshop was conducted in each district to introduce the future trial, and to explain the purpose of the rapid assessment and elicit initial feedback on the plans. Head teachers from each of the 75 schools eligible for the rapid assessment were invited to the workshop. The rapid assessment data collection was conducted between May-October 2021. The methods were based on, and adapted from, participatory rapid appraisal approaches [25] specifically using institutional and social mapping and transect walks to observe the WASH and the menstrual health physical and social environment of the schools. We also used a combination of semi-structured interviews, group discussions and structured observations [26]. Central to these methodological approaches was observation: spending time in the school communities, discussing with teachers, students and parents as well as learning about the school environment.

### Study participants

At each of the 75 schools, approximately 12 participants were selected as representative of the school community in terms of sex, occupation and education status. Participants were students aged 15–24 years and staff. The aim was to collect enough in-depth data from individuals and groups to help us capture variations in informants’ perspectives and experiences related to the study research questions as outlined in Table 1.

**Table 1:** Rapid qualitative assessment: Research activities, objectives, questions, methods & participants as per initial protocol.

Research activity	Objective	Key research questions	Methods	Participants
**Interview with head teachers or senior teacher**	To explain the purpose of the exercise, manage expectations and concerns and collect initial data.	Is the school eligible and willing to participate in a future trial? Does the school have the 2015 Government MHM Charter? Are any policies, programmes and activities related to menstrual and reproductive health used at the school? If so, which? What WASH and illness management facilities are available at the school? What management structures are in place in the schools (deputies, senior male teachers, school governance body, Parent Teachers Association (PTA), directors, committees etc.,)? How has COVID has influenced the school dynamic? Have any new COVID policies or guidelines been implemented in the school?	Semi-structured interviews to understand current and future school programmes.	School head teacher (or delegated senior teacher). (N = 75)
**Transect walk with WASH observation**	To observe the social and physical environment of the school, relevant to menstrual health.	What is the school setting? Does the school have basic WASH facilities? How many toilets blocks are there for girls and boys respectively? What are the facilities to manage minor illness on site? Are painkillers and menstrual materials available at the school? Are there posters on the walls, or other indicators related to reproductive health programmes?	Transect walk starting from central point, moving in concentric circles around the school and taking care to stop, listen, look and chat on the way. A rough sketch map of the setting, showing particular features associated with the WASH environment and facilities to support illness. Structured WASH checks to describe WASH access (access to water, access to separate gender blocks, type of sanitation facilities, access to hand-washing stations).	A wide range of the school community were engaged in informal conversations during the walk. Teachers, and/or student representatives acted as guides. (N = 75)
**Student group discussion**	To hear student perspectives of the social and physical environment of the school, relevant to menstrual health.	Description of the school environment (including WASH and illness management). Perceptions of the interventions, support structures and facilities related to WASH and reproductive or menstrual health in the school. What COVID-related interventions are taking place in schools? What activities and groups or committees did students take part in?	Group discussions	2 group discussions (GD) in each school. 4 participants in each group. ▪ 1 GD female students ▪ 1 GD male students. (N = 600)
**Semi-structured interviews**	To gain a deeper understanding of perspectives of the social and physical environment relevant to menstrual health.	Description of the school environment in relation to menstruation and illness management. What groups or committees existed in the school and what were their roles? How are WASH facilities maintained at the school? Perceptions of the interventions, support structures and facilities related to WASH and reproductive or menstrual health in the school or in the wider community. Recommendations for introducing and delivering new activities and programmes in the school. Any further issues raised in the GDs and IDIs.	Semi-structured interview covering key themes, as above and building on themes raised in the group discussions.	3 at each school - Senior teacher (N = 75) - One female student (N = 75) - One male student (N = 75)

### Participant recruitment

The research team reached out to the administrator of each school to obtain a register including the contact details of the parents of 32 students (16 boys and 16 girls) aged 15 years and above. From the lists provided by the school administration, the research team randomly selected 10 students (5 boys and 5 girls) to participate in the rapid assessment activities (Group Discussions (GDs) [1 GD for each gender with 4 participants] and in-depth interviews (IDIs) [1 IDI for each gender]).

**Pre-COVID-19.** The research team through the school administration sent out invitation letters requesting the parent or guardian of the selected students below 18 years to come to the school premises to obtain study information and provide written parental consent if they agreed for their student to participate in the rapid assessment activities. The research team obtained written assent for the students below 18 years if they agreed to participate in the study. Additionally, students above 18 years were provided with the study information and those who agreed to participate in the study provided written informed consent.

**During COVID-19.** Due to the COVID-19 related restrictions to conducting in-person fieldwork, some activities, especially participant recruitment, were conducted by telephone. The team contacted the parents of the selected students through a phone call for parental consent. Upon verifying the details of the parents against the school administration register, the research team provided the study information and asked the parents to provide verbal parental consent for those students below 18 years if they accepted for their student to participate in the study. Thereafter, verbal assent was obtained from the student if they accepted to take part in the study. Both the verbal parental consent and student assent were documented in the consent notebook. Additionally, students above 18 years were directly contacted using the phone details provided by the school and verbal consent was obtained and documented in the consent notebook for those who accepted to take part in the study.

### Data collection

Prior to data collection, the research team (13 male and 12 female researchers) were trained to facilitate and administer the sets of research activities as described in Tables 1 and 2. The team was trained on conducting face-to-face and telephone interviews and seeking written and verbal consent and/or assent as appropriate in line with the COVID-19 guidelines.

**Table 2:** Data generation strategies used during the study pre- and during COVID-19 restrictions.

District	Wakiso		Kalungu		
Research method	Pre-COVID-19 Restrictions	During COVID-19 Restrictions	Pre-COVID-19 Restrictions	During COVID-19 Restrictions	Total
Group discussion	18	0	8	0	26
In-depth interviews with teachers	20	94	6	30	150
In-depth interviews with students	18	185	6	65	274
Transect walk and school WASH observation	11	0	2	0	13

**Fig 1:**
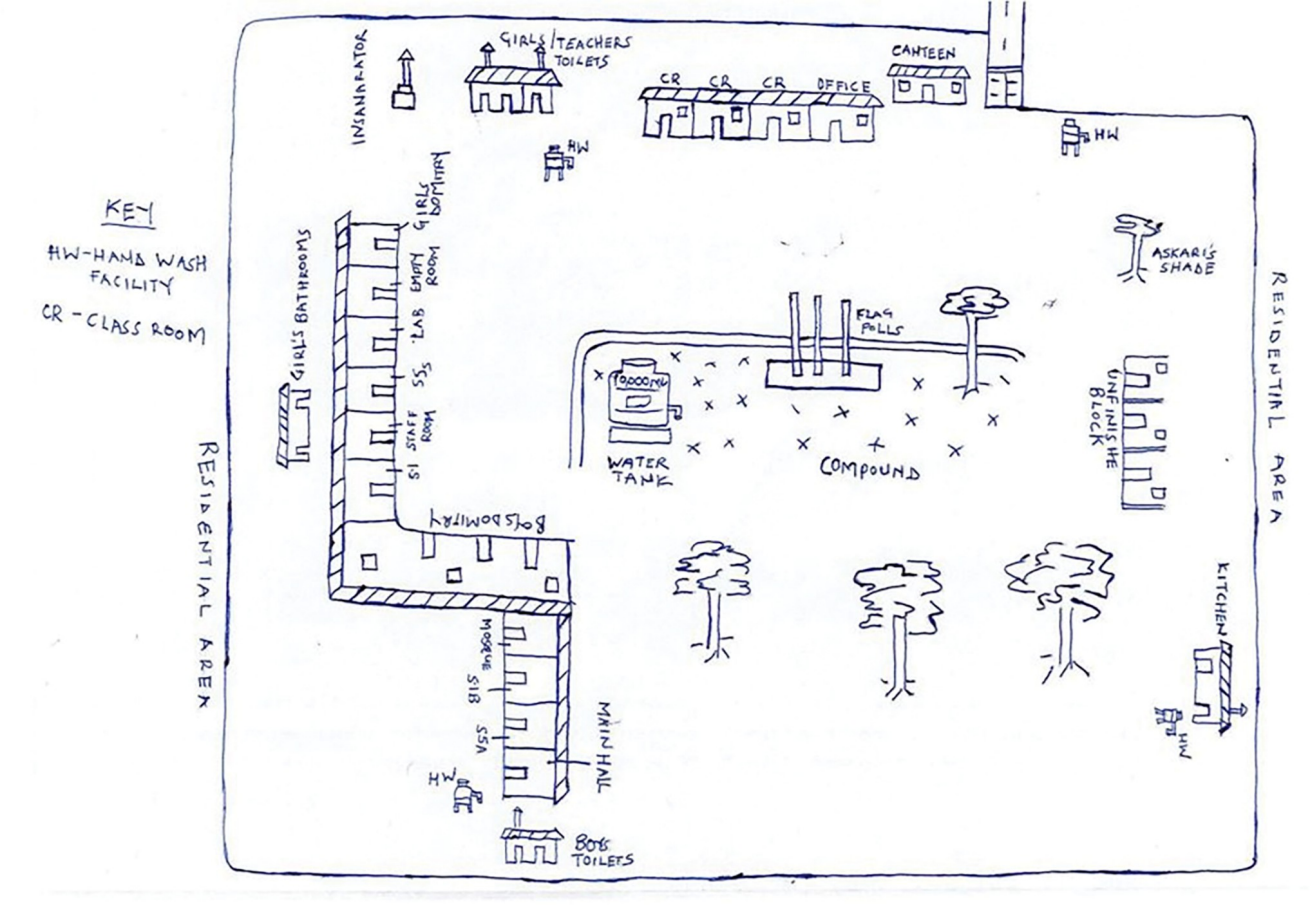
Transect walk sketch map.

We initially planned to conduct two IDIs with the head teacher and senior teacher, two IDIs (one female and one male student), two GDs (one male and one female GD of 4–6 participants in each school), and one transect walk with structured observation in each school. The transect walk following a guide (S1 Text) involved structured observations and a map (Fig 1), guided by an observation checklist (S2 Text). The observations focused on the school basic WASH facilities (number of sex-specific toilets blocks, access to water and soap, access to disposal methods), facilities to manage menstruation and minor illness and other indicators related to reproductive health programmes.

Before the COVID-19 lockdown (22nd May and 6th June 2021), we conducted face to face activities which included 50 IDIs with head/senior teachers and students, 26 GDs with 4–6 students stratified by age group and gender to encourage open discussion using semi structured interview guides (S3–S7 Text) and 13 transect walks with structured observations as schools were open. On 7th June 2021 the Presidential directive was given to close all schools for 42 days during the second wave of COVID-19. However, schools remained closed until 10th Jan 2022. Additional restrictions were put in place such as, public transport being suspended for 42 days, inter-district travel ban and public gatherings suspended. This disallowed the movement of the research team to the schools to conduct face-to-face data collection. Following the COVID-19 restriction, the study protocol was amended and approved by UVRI-REC (Ref-No. GC/127/816) to allow for the conduct of phone interviews as an alternative mechanism for data collection. The IDIs were replaced by phone interviews and each GD was replaced with two phone IDIs with students per school using phone interview guides (S3–S7 Text) (Table 2).

### Data analysis

Data analysis was done in two main stages—rapid analysis and a subsequent finer analysis. For the rapid analysis, detailed IDI and GD scripts were made by the interviewers. Regular meetings were held to generate emerging themes and meaning of the codes throughout the data collection period. We employed thematic framework approach [27] using the SEM for data analysis [19]. To achieve data familiarity, seven researchers and a lead analyst read the scripts several times and generated a codebook and themes based on the qualitative data collection guides (S3–S10 Text). Twenty-four scripts were initially coded manually to generate initial codes, emerging and recurrent themes. At this stage the remaining transcripts were coded using a refined codebook. The finer analysis happened after the initial rapid assessment report was completed, and summary findings shared with the participating schools and the study stakeholders in a dissemination workshop.

### Ethics statement

Participants were asked for written informed consent if aged ≥18 years, or for written informed assent and parental informed consent if aged <18 years. Due to COVID-19 restrictions on gatherings and face-to-face meetings, informed verbal consent/assent was obtained over the telephone from the study participants prior to participating in telephone interviews. All data were stored on encrypted computers and uploaded to the MRC/UVRI and LSHTM Unit secure server which is accessible only to selected members of the research team. Field notes and signed participant-informed consent were kept in a locked drawer at the study site. Participants were given unique identifying numbers; names and other school or participant identifying information were not recorded in the transcripts to ensure confidentiality.

Ethical approval was granted by the Uganda Virus Research Institute Research and Ethics Committee (February 22, 2021; GC/127/811); Uganda National Council for Science and Technology (March 17, 2021; HS1270ES) and London School of Hygiene and Tropical Medicine (LSHTM) Ethics Committee (March 23, 2021; 22951).

## Results

A total of 150 IDIs with the head/ senior teachers, 274 IDIs with students, 26 GDs (8 GDs in Kalungu (females n = 4, males n = 4), 18 GDs in Wakiso (females n = 9, males n = 9) and 13 transect walks with structured observations were conducted in the 75 schools (Table 2).

**Fig 2:**
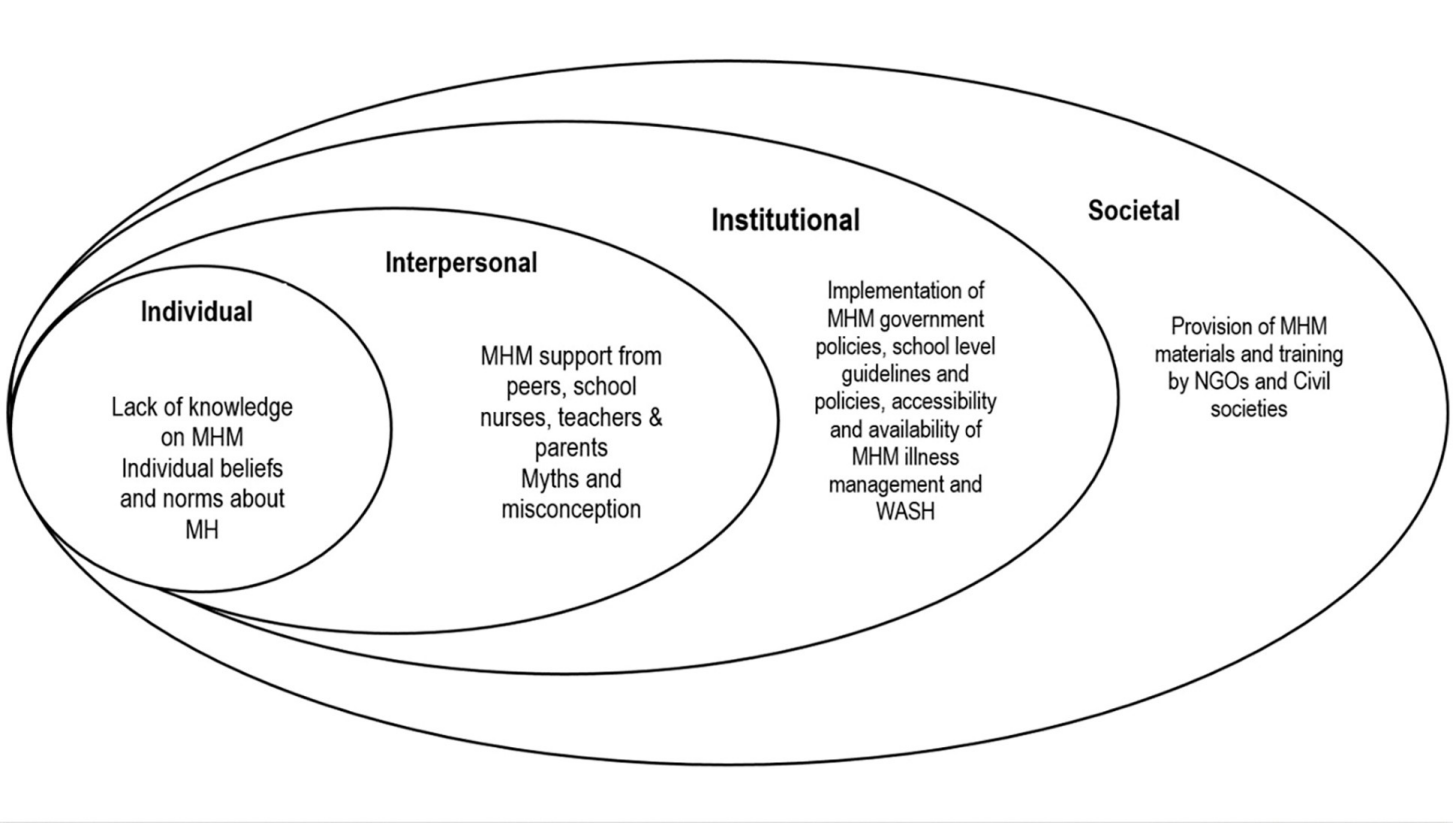
Illustration of the SEM framework showing the interrelations at various levels (adapted from [20]).

During the analysis, we identified themes describing social and physical menstrual health environments in secondary schools. These were categorised into four levels (individual, interpersonal, institutional and societal) based on the SEM (Fig 2).

### Individual level factors

#### Knowledge gaps on menstrual health

Students mentioned their limited knowledge about menstrual health, and some did not know what to do when they first experienced their periods, including not knowing the menstrual absorbent material to use. There were reports of young girls crying when they experienced menstruation as they did not know how to manage the situation.

*“There is ignorance among girls in the community (at home) and even at school on the different issues related to menstrual hygiene management*. *Most girls in the community use clothes to pad themselves because they don’t know the right materials to use and have no information related to menstrual hygiene management … they don’t know much about menstruation and sometimes look worried*.*”* (Female IDI 17 years)

For some of the girls who used cloths to manage their menstrual flow, they were not knowledgeable about the proper management of these materials in terms of washing, drying, and storage.

Some male students had limited knowledge of menstrual health because they had not received any menstrual health training at school or in the community. Training at school and community level organised by different agencies was designed only for girls. Some male students wanted to address this situation and proposed being involved in future menstrual health trainings to empower them with information to support the girls.

*“Most of the male students in the school are ignorant about menstrual issues because most of the organized trainings are for girls*. *We are never involved yet we relate with them in many ways*. *It’s important for the boys to learn about menstrual health because it gives them more knowledge on how to help the girls during their menstruation*.*”* (GD Male students)

Additionally, the management of menstrual pain was affected by students’ limited knowledge about analgesics, and the widespread belief that analgesics were not effective. Some students preferred using other methods like taking warm water, using herbs and exercising to manage the pain.

*“I believe the painkillers given at school for menstrual pain relief are not effective because girls complain that even after using the painkillers*, *the pain does not stop at all*. *Many girls now resort to using herbal medicine to manage their menstrual pain*.*”* (Female IDI 16 years)

#### Beliefs and norms about menstrual health

Teachers and students stated that girls hesitated to discuss menstrual issues with the boys and men, due to misconceptions about menstruation and social norms which caused shame and the fear of being mocked, judged, viewed as ready for sex or marriage and feeling like it is a punishment.

*“Sometimes girls have menstrual secrets that they cannot share with boys or men because they can easily make fun of them or take them as ready to conceive*. *So issues concerning menstrual health and reproductive issues among girls are kept a secret and not shared*.*”* (Male IDI 15 years)

The female participants further explained that menstruation is a personal issue that should not be discussed. Some female students referred to menstrual periods as stomach pain to keep it confidential even to a fellow female.

*“Students believe that menstrual issues should not be disclosed to anyone even to a fellow woman*. *When I go to inspect in their dormitory and find them sleeping during class time*, *they will say I have stomach ache or my stomach is paining to mean menstrual periods*.*”* (Senior woman teacher)

*“Sometimes I make inspections around the school and I find girls crying outside the classroom block or behind the toilet block*. *When I ask them if there is any problem*, *they always tell me it is stomach issues*. *They will never tell you that it’s menstrual periods*.*”* (Senior male teacher)

Other girls reported ‘hating’ their periods and that time of the month when they are menstruating as this make them feel uncomfortable and worried about staining their skirts with blood while associating with their male counterparts or during class time.

*“Some girls say they hate menstruation because it makes them feel uncomfortable sitting with boys at school especially those with the heavy flow because they are worried about bloodstains on their skirts*. *Some girls are really shy*, *they fear and will never ask a colleague for help during their menstrual periods*, *so they suffer in silence*.*”* (Female IDI 17 years)

The perception towards menstruation categorised as ‘hate’ and ‘fear’ has implications for girls’ confidence, self-esteem and relationships with their fellow students during puberty. The girls’ lack of self-esteem during menstruation also ‘*affects other reproductive health issues where so many girls have been left behind*, *cannot speak out what they are going through because they don’t believe in themselves*.*’* (Senior male teacher)

#### Poverty

Students and teachers agreed that girls from households that are economically unstable have limited capacity to afford decent, safe and appropriate menstrual products.

*“Some students come from poor families and their parents can’t afford to buy the pads that they can use for the entire term*, *some end up using nothing at all and struggle during their periods*.*”* (Female IDI 18 years)

During the interviews, many of the school senior teachers and head teachers made recommendations for additional stakeholders to support their schools ‘in the fight to keep girls in school’ chiefly by providing MHM materials.

“*My call is for responsible stakeholders to come on board and support students with menstrual pads*, *better WASH facilities and also training them how to make reusable pads or providing them with the materials for making the reusable pads so that they can start making their own pads*.*”* (Senior woman teacher)

### Interpersonal level factors

#### MHM support from member within the school

In some schools, students reported receiving psycho-social support, painkillers for pain relief, counselling and guidance in menstrual and reproductive health from members within the school. School nurses and senior teachers were reported to provide this support and information to students experiencing challenges on how to manage their menstruation.

*“The school has a senior woman teacher and a school nurse who conduct counselling and guidance sessions*. *They encourage girls to bathe*, *be clean*, *take warm water to relieve menstrual pain*, *and sometimes give out pain killers to those experiencing severe menstrual pain*. *They guide those who have not menstruated about what they are supposed to do and how to pad themselves*.*”* (Female IDI 18 years)

Participants shared that applying heat to the abdomen by placing a hot water bottle against the abdomen to relax the muscles and relieve cramps is a tool to manage menstrual pains, although most girls did not have access to a hot water bottle. In addition to giving psycho-social support, senior women teachers equip girls with the relevant information on how to make reusable sanitary pads. However, students revealed that some school nurses and senior teachers were “rude” and therefore the girls could feel uncomfortable approaching them.

“*We fear to approach teachers when we have problems and emergencies concerning menstruation because they are not friendly*. *Therefore*, *we urge teachers to offer parental care and support to students while at school*.*”* (Female IDI 16 years)

Female students approached the peers they trusted and shared their menstrual challenges, for support such as accessing menstrual materials to use in case they did not have.

*“Female students help one another during times of menstruation*. *If a girl needs a pad and maybe she does not have the money to buy one*, *she can borrow from a friend*.*”* (Female IDI 15 years)

Some male students recognized the importance of empowering the male teachers with menstrual health information so that they are confident to address menstrual health, leading to a less stigmatising environment in schools.

*“Male teachers need to be informed and have confidence regarding menstruation and menstrual hygiene so that they can support female students instead of laughing and mocking them to create a less stigmatizing environment at school*.*”* (Male IDI 17 years)

#### Managing the disposal of sanitary materials

Another important aspect raised during the study related to the disposal of used sanitary materials. Only 16 out of the 75 schools assessed in the study reported having incinerators where they dispose and burn the used sanitary materials. However, some female students reported being prohibited by their parents from disposing of the used menstrual pads in the school toilets or incinerators but instead carried them home for fear of the material being used to bewitch them.

*“Witches use menstrual blood to cast spells on a person and one way to get this blood is from the menstrual materials*. *My mum told me never to dispose my pads at school or anywhere*. *So I pack my used pads in a polythene bag and take them back home because I do not want to be bewitched*.*”* (Female IDI 17 years)

### Institutional level

#### Availability and implementation of the Government MHM charters and circulars

Only 4 of the 75 schools had the 2015 Government MHM Charter available, and 20 of the 75 had the MoES Circular. The Charter and Circular were not being implemented effectively in these schools. A head teacher commented: *“The school received one copy of the 2015 Government MHM Charter a while ago but I don’t know whether the school director uses it or where it is for us to action*.*”*

The remaining schools had not heard of or received the Government Charter or Circular. Most MHM policies are better accessed and recognised in government than in private schools and this leaves the latter lag behind in terms of implementation of the MHM guidelines.

*“Most of the times*, *the government programs are not well promoted and easily implemented in private schools*. *Those policy documents you are talking about are well accessed in government schools and they receive support to implement them but not our private schools*.*”* (Senior woman teacher)

Teachers reported that the MHM policy frameworks and information materials available to them need to be updated. Some teachers reported that the information materials issued to the schools are torn and worn out since few copies were provided. They recommended that the guidance should be provided in brochures or magazines so as to be easy to read and to keep.

#### School-level guidelines and policies

Menstrual health related activities were ongoing in six out of the 75 schools, run by civil society groups, health workers, and alumni school associations. The ongoing menstrual health activities were embedded in the general counselling sessions for students. Many of these activities focused on conducting puberty and reproductive health education, guidance and counselling, WASH-related talks for all students and menstrual health education for the girls.

*“We have meetings to discuss topics to do with puberty*, *WASH*, *especially regarding maintaining the latrines in the school*. *Some of them misuse the sanitation facilities*, *some use their hands*, *instead of toilet tissue*.*”* (Head teacher)

For such schools it was part of the school program to hold meetings with girls to discuss issues like menstruation, and reproductive health. Ten of the 75 schools encouraged girls to carry enough disposable sanitary materials but not re-usable materials as part of their school requirements.

*“On the side of the girls*, *they are encouraged to bring enough sanitary towel however*, *they should not be re-useable*. *This is done to avoid stigma among the fellow female students*. *Secondly they don’t have space where students can hang them after washing*.*”* (Senior woman teacher)

Conversely, in a few schools, male students reported that ‘*girls carry whatever they can use as sanitary materials and it is known that girls use pieces of cloth and others use re-usable pads’*. However, it was not clear what source these students had for this information. For some schools, students who needed extra menstrual materials during the school term were encouraged to approach the senior woman teacher or nurse to get some emergency pads to use.

#### Easy access to WASH and illness management facilities

Thirty-eight out of 75 schools reported having furnished sickbays with qualified nurses knowledgeable about managing menstrual related problems while others utilised community health facilities. However, some female students mentioned concerns with the attitudes of school nurses which limited their ability to access medical attention.

*“The nurse is always rude and tough on the girls*. *Even when you experience severe cramps and need medical attention*, *the nurse will always say you are pretending and in the end*, *you struggle with your pain and are not helped*.*”* (GD female students)

The water sources accessed in the different schools varied: Twenty-one out of 75 schools reported access to both pumped water from protected community wells and underground water, 44 schools reported harvested rainwater stored in the tanks. Thirty schools school reported having functional boreholes and piped national grid water. In some schools, the available water source was only accessible during particular school times which affected access to water among female students especially when they needed to change their menstrual material when they were menstruating.

*“We have a water tap in the school compound which supplies the entire school but sometimes it has no running water and this affects us during the menstrual periods*. *We cannot access water when we want to change our pads and clean our bodies*.*”* (GD female students)

Sixty-seven of the 75 schools did not have private changing rooms and the girls opted to use the toilets in case they needed to change their menstrual materials.

*“The girls don’t have a private room to change their sanitary pads*. *They improvise from the toilets and bathrooms to change*.*”* (IDI female student)

However, the lack of privacy, lack of water and anal cleansing material, lack of cleanliness and menstrual absorbent disposal systems within the toilet cubicles discouraged female students from using the toilets, expressing fears that dirty toilets posed risks of diseases and infections leaving them few options during their menstrual periods.

### Societal level

#### MHM support from NGOs and old students’ associations

Many of these MHM programmes, where they existed in schools (see above), focussed on provision of sanitary materials, training girls and parents on how to make re-usable sanitary materials, provision of puberty and reproductive health education and conducting sensitisations on hygiene and sanitation.

*“There is an NGO that has played a very big role in providing sanitary materials for the girls but also teaching both the parents and students in making reusable sanitary towels*. *This same organization also gives girls information about puberty and reproductive health*.*”* (Head teacher)

Schools found these menstrual health education programmes valuable to provide students and teachers with menstrual health knowledge as well as support to rapidly integrate menstrual health into existing programmes.

## Discussion

We found limited support for menstrual health and little knowledge about menstrual health among girls and many of those working in schools in Ugandan secondary schools, despite the existence of national level support for MHM in schools. Girls were reluctant to discuss menstrual health issues with teachers, any male gender and some of their fellow girls including not seeking psycho-social support. There were reports of some schools prohibiting the use of re-usable sanitary pads, limited access to safe water during school hours, unclean and not private toilets and poor disposal systems for sanitary pads. The poor menstrual environment could lead to menstrual-related school absenteeism and low completion rates for girls as reported in previous studies [13,28,29].

At the individual level, girls reported negative experiences related to menstruation such as crying, hating their periods since they make them feel uncomfortable and getting worried, which affected their self-esteem and confidence with their fellow peers. Similar results were observed in a systematic review and qualitative meta-synthesis of women’s and girls’ experiences of menstruation, where girls perceived menarche as something to be feared [1]. This may lead to anxiety, depression and a lack of preparedness on how to handle menstruation [30,31]. Additionally, our findings showed the limited knowledge and the unmet need for engaging boys and male teachers, who have the potential to support females in MH. Increasing male involvement in supporting menstrual health minimizes stigma, teasing and embarrassment thereby improving the social school MH environment and overall physical and mental health of the adolescent girls.

The limited knowledge on menstrual health has been largely compounded by cultural norms and beliefs that menstruation is secret. Participants described menstruation as a girls’ or women’s issue that should not be discussed because of the fear of negative consequences. A finding consistent with previous studies [32,33]. Indeed, other research has indicated that there are limited opportunities for young women to discuss menstruation openly [34,35]. Menstrual health interventions for puberty education that address the cultural beliefs and educate men around issues of MH are warranted.

Participants’ limited access to financial resources affected their affordability to safe and appropriate menstrual products. Economic vulnerability makes many school going girls unable to access adequate menstrual products and pain management mechanisms that are costly forcing many girls to resort to unhealthy coping mechanisms [35–39]. Many girls resorted to using alternative materials like pieces of cloth, cotton or toilet paper. This finding is similar to previous studies among school-going girls that found that menstrual problems (lack of access to menstrual products) are linked to poverty [35–37], and implies that in-school girls should be supported with access to and training on use of menstrual management materials or products. Access to safe materials to manage menstruation has been found to be associated with reduction of urogenital infections [38,39].

At the interpersonal level, we found that while some girls did receive menstrual health and reproductive health information from the teachers, their female peers often played a key role in supporting each other at school by helping each other find menstrual supplies or covering up each other’s menstrual accidents if they occurred. Most of the girls found it comfortable to seek support from their peers and therefore peer support embedded in menstrual health interventions would improve the social menstrual environment in schools. Peer support can be used to sensitize fellow classmates about MH and has an impact on menstrual management behaviours. Similar results were reported in studies conducted in Uganda and Nepal where parents, siblings, peers, and teachers were sources of support and comfort to the girls experiencing their periods [2,40]. This support is critical and girls with peer support tend to have better MHM [41].

The current study identified that some female students practiced culturally restrictive menstrual management practices where female students are prohibited by their parents from disposing of the used menstrual pads in the school toilets or incinerators but instead carried them home for fear of being bewitched. Because of the fear of being bewitched, girls found it safe to pack their used pads and dispose them at home.

The cultural and restrictive beliefs led to the poor outlook on menstruation, unhygienic menstrual practices such as refraining to change menstrual materials while at school that can increase the risk of infections affecting adolescent health and the capabilities of girls in managing menstrual hygiene. Similar results were reported in a qualitative study where the disposal method was said to be determined by the belief systems of the family with some families believing that one’s blood should never be seen by others or the person would be bewitched [42]. Reproductive health challenges are reported to be significantly higher among women who practiced cultural menstrual restrictions [43]. We recommend providing adequate menstrual health information and sensitization on the appropriate MHM practices in schools. This is still limited in our setting but will importantly facilitate informed decision making in relation to menstrual hygiene management [9].

At the institutional level, the study revealed different government guidelines and policies being implemented and these include the National Sexuality Framework [44], the MHM 2015 government charter [15], the MoES circular, and the MHM reader Secondary schools. While we found that some schools were implementing Government MHM policies and guidelines, they were not necessarily implementing all of the guidance or doing so consistently. These results support the International Water and Sanitation Center study conducted in 2018 which indicated that 30% of the primary and secondary schools assessed in rural and urban areas were implementing some of the activities suggested in the MoES Circular through provision of changing rooms, emergency pads and the purchasing of handwashing [16,45].

A key finding from the study revealed that some schools restricted students on the type of menstrual products to use. Some schools prohibited the use re-usable sanitary pads. School authorities had concerns that girls may not have the ability to properly wash, dry, and care for the reusable cloth pads and worried that girls would share these pads, which if not cleaned properly cause vaginal infections. It is imperative to note that for girls in rural schools with little access and finances to procure disposable sanitary pads, reusable pads would be an important alternative.

Access to basic WASH facilities are essential for proper menstrual health management as it helps girls to manage their periods safely and keep in school [46]. From our study, in some schools where basic WASH facilities were available, students had restricted access to water sources and menstrual absorbent disposal systems and most of the toilets lacked privacy. These findings are consistent with the joint WHO UNICEF report 2018 showing that half of the schools in low income countries lack adequate water, sanitation, and hygiene services crucial to enable girls and female teachers to manage menstruation [47].

This finding was consistent with a formative study conducted in six schools located in rural areas in Mumbwa and Rufunsa districts in Zambia were girls preferred to stay home during menstruation for fear of the toilets at school that were usually unclean and lacked privacy [48]. Therefore, they chose not to use the toilets to manage their menstruation while at school. Similar results were reported in school WASH evaluation in Kenya that indicated girls were less absent or likely to drop out in primary schools when there were satisfactory private and clean wash facilities [49]. From the finding it indicated that providing schools with latrines which are built to accommodate menstruating girls’ specific needs for privacy, space, washing practices and correct disposal and/or cleaning of menstrual pads will lead to dignity and better attendance and thus improve girls’ access to education [50].

A limitation of our study is that due to COVID-19 restriction we were unable to conduct all the transect walks to observe the social and the physical environment. Therefore, data from the 13 out of 75 transect walks we conducted cannot be generalizable. At the time of the study, the school physical environment changed as a result of the new COVID-19 prevention measures that included and not limited to additional hand wash facilities and availability of soap and hand sanitizers, which may have meant that the WASH facilities observed did not represent a usual time. Another limitation was that in 62 of the 75 schools, we had to replace the 2 GDs with 4 IDIs which may have affected the nature of the information collected. However, the conduct of 2 IDIs Vs. 1 GD elicited more in-depth data, providing a greater volume of data to capture variations in informants’ perspectives and experiences for analysis. This was an amendment to the original protocol in order to comply with COVID-19 guidelines.

## Conclusion

This study suggests that the individual, interpersonal, institutional and societal burdens related to menstrual experiences affect menstrual health among young people in secondary schools in Wakiso and Kalungu districts, Uganda. The main menstrual challenges among female students reported are largely inadequate preparation for menarche, being unable to share or seek assistance, and having a poor and inadequate physical environment to support MHM at school. It is imperative to continue raising awareness on menstrual health among male students as this may change the negative sociocultural beliefs, misconception and stigma in the community surrounding menstrual health, creating a more favourable environment for the female students. Therefore, multi-level and evidence-based MH interventions could prepare girls for menarche, help to improve menstrual health and the social wellbeing of girls at all levels of the socio-ecological framework.

## Supporting information

S1 Text Transect walk guide. (PDF)

S2 Text Observation checklist. (PDF)

S3 Text Interview guide for headteachers. (PDF)

S4 Text Group discussion guide for students. (PDF)

S5 Text Interview guide for male students. (PDF)

S6 Text Interview guide for female students. (PDF)

S7 Text Interview guide for senior teacher. (PDF)

S8 Text Telephone interview guide for female students. (PDF)

S9 Text Telephone interview guide for male students. (PDF)

S10 Text Telephone interview guide for senior teachers. (PDF)
