# Supplementary material for: Understanding the social and physical menstrual health environment of secondary schools in Uganda: A qualitative methods study
Source: PLOS Glob Public Health. 2023 Nov 29;3(11):e0002665. doi: 10.1371/journal.pgph.0002665 (PMC10686490; doi:10.1371/journal.pgph.0002665)
Supplement: S1 Text — (PDF) [file pgph.0002665.s001.pdf]

Annex 2. Draft Transect Walk for the MENISCUS Rapid Assessment

Standard Operation Procedure

Version: **Draft V1**

Issue Date: **16/12/2020**

School code:

Facilitator: **Signature:**

....

Approved by: **<Approver>** **Signature:**

....

1. PURPOSE

The purpose of this SOP is to explain the procedures for conducting a transect walk around the school.

2. SCOPE

This SOP covers the process and procedures of conducting a transect walk.

3. POLICY AND PROCEDURE

3.1 Policy

3.1.1 The team leader will assign the roles and responsibilities of the team members for the session a week in advance giving them time to prepare and contact the local head teachers, to explain the purpose of the exercise and what the team will do.

3.1.2 The transect walk is a creative method which involves walking in a circular (spiral) path from a central point in the school towards the edge of the school while observing daily school activities, observing school settings and talking to members of the school.

3.1.3 The information collected during the transect spiral walk is used to provide background information school settings and environment. This information may inform the topic guides used for short interviews and community group discussions.

3.1.4 Each session will have two team members present for the spiral walk.

3.1.5 Each session will have a different number of participants, because it depends upon who the team meet as they move around the school.

3.1.6 Team members should take with them:

3.1.6.1 notebook and pen/pencil,

3.1.6.2 information sheets about the study, so that they can explain the purpose of the exercise

UGANDA VIRUS RESEARCH INSTITUTE ENTEBBE
RESEARCH ETHICS COMMITTEE
APPROVED
VALID UNTIL
22 FEB 2022
P. O. BOX 49, ENTEBBE
UGANDA

3.1.6.3 Any materials required by the Risk Management Plan for mitigating risk of COVID transmission.

3.1.7 Any problem encountered at a site, of a work or personal nature, must immediately be reported to the team leader for problem-solving.

3.2 Conducting the spiral walk

3.2.1 The team will consult head teachers or equivalent to gain a rough idea of the layout of the school.

3.2.2 Based on this information the team must decide on the path that should be taken to cover the full geographical variation in the area. (Note, the path is not a straight line from one side of the school to another. As far as possible the team should try and walk in every increasing circles so they cover a sample of areas across the schools and by the end of the exercise have a good idea of the layout of the place)

3.2.3 Walk slowly and patiently and try to understand the physical features and aspects related to the layout of the school from different perspectives. Ask people you meet to explain what happens in different places.

3.2.4 The team members may make rough notes as they go along and should write these notes up into a detailed narrative after the walk. They should be written up when they return to the office immediately to ensure nothing is forgotten.

3.2.5 The spiral walk will have raised school community's curiosity about what the team is doing. A small discussion on what the team has seen during the walk, may be helpful at the end of the walk with school community who may have gathered to find out what is going on, to help to assuage their curiosity

3.3 The map

3.3.1 The map is drawn from the team members during the walk. The aim is to produce a rough map to inform the wider team about school boundaries, the places where the classrooms and offices are found, the areas where latrines blocks are found, the place/s where water sources are located, the places where illness management rooms are found. The team can also include approximate distance between toilet blocks and more proximate water sources. In case of mixed day/boarding schools, the map can show where the bedrooms are in related to the classrooms. The map should be rough and act as a visual complement to the detailed notes written up from the spiral walk and the group discussion

UGANDA VIRUS RESEARCH INSTITUTE ENTEBBE
RESEARCH ETHICS COMMITTEE
APPROVED
VALID UNTIL
22 FEB 2022
P. O. BOX 49, ENTEBBE
UGANDA
