# Supplementary material for: Understanding the social and physical menstrual health environment of secondary schools in Uganda: A qualitative methods study
Source: PLOS Glob Public Health. 2023 Nov 29;3(11):e0002665. doi: 10.1371/journal.pgph.0002665 (PMC10686490; doi:10.1371/journal.pgph.0002665)
Supplement: S2 Text — (PDF) [file pgph.0002665.s002.pdf]

Annex 3. Draft WASH spot checks for the MENISCUS Rapid Assessment study

Standard Operation Procedure

Version: **Draft V1**

Issue Date: **16/12/2020**

School code:

Facilitator: **Signature:**

……………………………………………

Approved by: **<Approver>** **Signature:**

……………………………………………

1. PURPOSE

The purpose of this SOP is to explain the procedures for conducting the WASH spot checks in each school for the MENISCUS rapid assessment.

2. SCOPE

This SOP covers the process and procedures of conducting a WASH spot check.

3. POLICY AND PROCEDURE

3.1 Policy

3.1.1 The team leader will assign the roles and responsibilities of the team members for the session a week in advance giving them time to prepare and contact the local head teachers, to explain the purpose of the exercise and what the team will do.

3.1.2 The WASH spot check is a method which involves observation and record information about the school WASH facilities. A series of questions directed to head-teacher (or other teachers) are also included to find out further information regarding WASH school information.

3.1.3 The information collected during the WASH spot checks is used to provide background information about school settings and environment. This information may inform the topic guides used for short interviews and community group discussions.

3.1.4 Each WASH spot check visit will be conducted by two team members.

3.1.5 Team members should take with them:

3.1.5.1 Tablets to complete the WASH spot checks template.

3.1.5.2 Information sheets about the study, so that they can explain the purpose of the exercise

3.1.6 Any problem encountered at a site, of a work or personal nature, must immediately be reported to the team leader for problem solving.

...SEARCH INSTITUTE ENTEBBE
RESEARCH ETHICS COMMITTEE
APPROVED
[illegible]
22 FEB 2022
P. O. BOX 49, ENTEBBE
UGANDA

3.2 Conducting the WASH spot check visit

3.2.1 The team will consult head teachers or equivalent to gain a rough idea of where the toilets blocks are located in the school and also the team will ask some initial questions related to water and sanitation access before moving to the completion of the WASH spot check tool:

SPOT CHECK TOOL:

Question for teacher:

WATER
1)What is the **main** water source at the school currently?
1: Piped water into school building 2: Piped water into schoolyard/ plot 3: Public tap/ standpipe 4: Tube well / borehole 5: Protected dug well 6: Unprotected dug well 7: Protected spring 8: Unprotected spring 9: Rainwater collection 10: Bottled water 11: Cart with small tank/ drum 12: Tanker truck 13: Surface water 14: Students bring water to school from their homes 15: No water available in/near school 88: Other

2) How far away is the school's current water source? 1: The current water source is on school grounds. 2: The current water source is ____metres away. 3: Water brought from home.

3) Is the main water source functional now? 1: Yes 2: No

SANITATION
5) Does the school have any toilet facilities? 1: Yes 2: No

6) How many toilet blocks does the school have? _____

7) What type of sanitation facilities does it have? (mark all the types found in the school)
-Flush/pour flush toilets,
-Ventilated improved pit latrines,
-Composting toilets
-Pit latrines with a slab or platform.

8) Are they gender segregated toilets blocks? 1: Yes 2: No

Spot Check Observation Tool: Team members will go and check all the following items:

9) Is the water source functional : 1: Yes 2: No

10) Write down all the type of water sources:
-piped water,
-borehole or tube wells,
-protected dug wells,
-protected springs and
-packaged or delivered water

UGANDA VIRUS RESEARCH INSTITUTE ENTEBBE
RESEARCH ETHICS COMMITTEE
APPROVED
VALID UNTIL
22 FEB 2022
P. O. BOX 49, ENTEBBE
UGANDA

11) Go and check if there are toilets facilities and check that the number of blocks are correct.

12) Write down the type of sanitation facilities:
-Flush/pour flush toilets,
-Ventilated improved pit latrines,

-Composting toilets
-Pit latrines with a slab or platform.

13) Check if there are gender segregated toilets blocks: 1: Yes 2: No

14) Check if at least one toilet is functional (accessible, functional and private (with at least 1 door).

15) Check if there are disposal pit or/and disposal bins or other disposal devices for menstrual products and write down where they are found (outside, inside cubicles, both…)

16) Other: The team members may make rough notes about anything interesting they found.

UGANDA VIRUS RESEARCH INSTITUTE ENTEBBE
RESEARCH ETHICS COMMITTEE
APPROVED
22 FEB 2022
P. O. BOX 49, ENTEBBE
UGANDA

UGANDA VIRUS RESEARCH INSTITUTE ENTEBBE
RESEARCH ETHICS COMMITTEE
APPROVED
VALID UNTIL
22 FEB 2022
P. O. BOX 49, ENTEBBE
UGANDA
