# Supplementary material for: Understanding the social and physical menstrual health environment of secondary schools in Uganda: A qualitative methods study
Source: PLOS Glob Public Health. 2023 Nov 29;3(11):e0002665. doi: 10.1371/journal.pgph.0002665 (PMC10686490; doi:10.1371/journal.pgph.0002665)
Supplement: S3 Text — (PDF) [file pgph.0002665.s003.pdf]

**Annex 1: Draft Semi-structured interview with school head teacher in the MENISCUS Trial**

**Date:** **Start time (24hr format):** **End time:**

**School name (District name):**

**Name of Teacher interviewed:**

**Facilitator's names:**

**Primary objectives:**

1) To explain the purpose of the exercise, manage expectations and concerns
2) Collect initial data on the school environment, implementation of menstrual health programmes, school committee structure and influence of COVID on the school.

| **Introduction of the project and purpose of the rapid assessment exercise**<br>Interviewer will introduce the study, the purpose of the exercise and will explain the activities that will be conducted in the next two weeks. | |
|---|---|
| **Opening Questions – Personal background and questions about school**<br>Opening questions are intended to build rapport and gradually lead into the key questions | |
| **Questions** | **Probes/follow-ups** |
| For how many years have you been a teacher? How many in this school? | |
| **Questions about basic school information** | |
| 1. School location: Rural___ Peri-Urban___ Urban___ | |
| 2. School level: Secondary__ Mixed School____ | |
| 3. Type of school:<br>Day school___ or Mixed Day/Boarding school____<br>or Boarding school ____ | *If the school is exclusively boarding, it should be excluded.* |
| 4. Number of students in:<br>Day school _______ Boarding School_______ | |
| 5. Number of students in S1:<br>6. Boys_______ Girls_________ | |
| 7. School Management:<br>Public___ Private____ Religious___ Other___________ | |
| 8. How many members of staff are employed at the school?<br>Full-time staff ___ Teaching staff_____ | |
| | |

UGANDA VIRUS RESEARCH INSTITUTE ENTEBBE
RESEARCH ETHICS COMMITTEE
APPROVED
VALID UNTIL
22 FEB 2022
P. O. BOX 49, ENTEBBE
UGANDA

| | |
|---|---|
| 9. How is the school managed? How involved are the school managers? | Probe for roles of deputies SMT/school governance body/PTA/directors. |
| 10. Does the school have access to basic sanitation and water access? | **(1) Access to drinking water**: Drinking water from an improved source* and water is available at the school at the time of the survey) **Improved water source: piped water, borehole or tubewells, protected dug wells, protected springs and packaged or delivered water.*<br><br>**2) Access to sanitation:** improved sanitation^ facilities at the school that are single-sex and usable (available, functional and private) at the time of the survey. *^Improve sanitation facilities: flush/pour flush toilets, ventilated improved pit latrines, composting toilets and pit latrines with a slab or platform.* |
| 11. Does the school have facilities or services for illness management? Which ones? | Probe for how students access these facilities or services and who is responsible for the facilities or services. |
| **Now we'll move to information about current and future programmes, policies and activities related to puberty and menstruation** | |
| 12. Does the school have a copy of the 2015 Government MHM Charter?<br><br>13. How is puberty and menstruation-related education taught at the school? (*Examples: As a component of the core curriculum, as a stand-alone special module on menstrual hygiene exclusively, through a school-sponsored extracurricular program etc.)*<br><br>14. Are any policies, programmes and activities related to puberty, menstrual and reproductive health used at the school? If so, which ones?<br><br>15. Do you know of any new programmes relate to puberty, menstrual health and reproductive health that are planned for the next year?<br><br>16. Are menstrual materials available for girls at the school on a regular basis? What materials are available?<br><br>17. Have teachers in the schools ever received any training or information on menstrual health? If so, when and what training? | Probe for current and future planned government programmes, NGOs activities, reproductive health programmes related to puberty (including vaccination for HPV.) |

| | |
|---|---|
| | |
| **Now we'll move to information about COVID related school changes and new programmes** | |
| 18. What challenges has COVID caused for your school?<br><br>19. Have you implemented any new government COVID guidelines? Which ones? Are there some guidelines related to WASH changes in the school environment? | Probe for specific changes in school functioning, separation, new school schedules, use of mask, more hand-washing stations? |
| **Explanation of plans for the MENISCUS trial and approval to participate**<br>Interviewer explains the planned MENISCUS intervention, it's components and expected timelines, explaining that 50% of selected schools will receive the intervention within the next year and the other 50% will receive the intervention after the study has been completed.<br><br>Allow time for participant to ask questions about the intervention. | |
| 20. Would your school would be willing to participate in the MENISCUS trial? | |

| **Closing Questions** |
|---|
| 1. Do you have anything else would like to add to the discussion that we have not yet covered? |
| 2. Do you have any questions for us? We may not be able to answer them all, but we can do our best. |
| **To end our discussion, I want to thank all of your time and speaking with us today. We really learned a lot from you!**<br><br>**Before we leave today, does anyone have opinion, ideas, or thoughts they would like to add or ask? Is there something you would like to talk about? Is there something we talked about today that you would like to know more about? Thank you for participating.** |

UGANDA VIRUS RESEARCH INSTITUTE ENTEBBE
RESEARCH ETHICS COMMITTEE
APPROVED
[illegible]
22 FEB 2022
P. O. BOX 49, ENTEBBE
UGANDA
