# Supplementary material for: Understanding the social and physical menstrual health environment of secondary schools in Uganda: A qualitative methods study
Source: PLOS Glob Public Health. 2023 Nov 29;3(11):e0002665. doi: 10.1371/journal.pgph.0002665 (PMC10686490; doi:10.1371/journal.pgph.0002665)
Supplement: S4 Text — (PDF) [file pgph.0002665.s004.pdf]

Annex 4. Draft Group Discussion Protocol Guide for students for the MENISCUS Rapid Assessment study

Date: **Start time (24hr format):** **End time:**

School code:

Facilitator's names

Primary objectives:

1) To hear student perspectives of the social and physical environment of the school, relevant to menstrual health
2) To explore functioning of existing students' committee at the school.

Participants

Conduct two group discussions (one with female students, one with male students) in each school with 4-5 participants in each group. Generate a list of participants through introductory meeting, transect spiral, local fieldworkers and observations in the community. Participants will be adolescents aged $\geq$15 years, who are considered representative of the school community.

Venue

A venue that offers privacy (free of distractions).

Seating arrangements

If possible, arrange the chairs in a semi-circle, without tables. Additional precautions should be taken to ensure social distance is maintained throughout the discussions. Place chairs 2 meters apart for each other, and provide participants with face masks and hand sanitizer prior to the group discussion. Always refer to the Risk Management Plan for the latest COVID guidelines.

Preparations

- Drinks and snacks for the participants and facilitators.
- Table of participant details pre-drawn on A4 paper to save time.
- Face masks and hand sanitizer
- All materials laid out and ready to use

Procedures

Participant characteristics

As you wait for participants to arrive, one facilitator should go around the circle, and, for each participant, record the following: Name, Age and Class. Please note this should be done by the facilitator, not filled in by participants themselves. This should be done before the introduction, with the exception of late comers.

UGANDA VIRUS RESEARCH INSTITUTE ENTEBBE
RESEARCH ETHICS COMMITTEE
APPROVED
22 FEB 2022
P. O. BOX 49, ENTEBBE
UGANDA

Introduction

We have selected you to represent your school community here as we really value what you as students can share with us about your experiences of the school environment. This information will help us understand the current status of the schools, access to WASH and illness-management facilities and menstrual health programmes being implemented in your school. This will help in planning ways of helping girls to become and stay healthier and complete studying at school through improved management of menstrual periods. Although we will not be providing money, we will be providing a drink and a snack during the discussion.

ADMINISTER INFORMATION SHEETS AND INFORMED CONSENT FORMS

- *Ask:* Which language are you most comfortable using (Luganda or English)?
- This will be a 1-2 hour activity.
- We want it to be as participatory as possible. We want to hear your views. There are no right or wrong answers.
- Please feel free to give your ideas and also give a chance to everyone to speak.
- You may have different ideas from others – that's okay – we want to hear them.
- Any question you feel uncomfortable about, please feel free not to answer it.
- Your names will be kept confidential – when we write up the discussion, we never use people's real names.
- We will be happy to answer any questions you have at the end of the discussion.
- **Is it okay with you that we start the discussion?**

**Topic Guide Themes for Group Discussions**
1. Description of the school environment (including WASH and illness management)<br>2. Perceptions of the interventions, support structures and facilities related to WASH and reproductive or menstrual health in the school<br>3. What COVID-related interventions are taking place in schools?<br>4. What activities and groups or committees do students take part in?<br><br>*Note: Topics could change or be revised following observations carried out during the rapid assessment.*

**Closing Questions**
1. Do you have anything else would like to add to the discussion that we have not yet covered?
2. Do you have any questions for us? We may not be able to answer them all, but we can do our best.
**To end our discussion, I want to thank all of your time and speaking with me today. We really learned a lot from you!**<br><br>**Before we leave today, does anyone have opinion, ideas, or thoughts they would like to add or ask? Is there something you would like to talk about? Is there something we talked about today that you would like to know more about?**<br><br>**Thank you for participating.**

UGANDA VIRUS RESEARCH INSTITUTE ENTEBBE
RESEARCH ETHICS COMMITTEE
APPROVED
VALID UNTIL
22 FEB 2022
P. O. BOX 49, ENTEBBE
UGANDA
