# Supplementary material for: Understanding the social and physical menstrual health environment of secondary schools in Uganda: A qualitative methods study
Source: PLOS Glob Public Health. 2023 Nov 29;3(11):e0002665. doi: 10.1371/journal.pgph.0002665 (PMC10686490; doi:10.1371/journal.pgph.0002665)
Supplement: S5 Text — (PDF) [file pgph.0002665.s005.pdf]

Annex 5B. Draft Semi-structured Interview Guide male students for the MENISCUS Trial

Date: **Start time (24hr format):** **End time:**

School code:

Facilitator's names:

Primary objectives:

1) To gain a deeper understanding of perspectives of the social and physical environment relevant to menstrual health
2) To explore functioning of existing students' committee and groups at the school.

**Topic Guide Themes for interview**
1. Background information about the informant<br>2. What activities and groups or committees do students take part in?<br>3. How are WASH facilities maintained at the school?<br>4. Perceptions of the interventions, support structures and facilities related to WASH and reproductive or menstrual health in the school or in the wider community<br>5. Any further issues raised in the group discussions with students<br><br>*Note: Topics could change or be revised following observations carried out during the rapid assessment.*

**Closing Questions**
1. Do you have anything else would like to add to the discussion that we have not yet covered?
2. Do you have any questions for us? We may not be able to answer them all, but we can do our best.
**To end our discussion, I want to thank you for your time and speaking with me today. I really learned a lot from you!**<br><br>**Before we leave today, is there something you would like to talk about? Is there something we talked about today that you would like to know more about?**<br><br>**Thank you for participating.**

UGANDA VIRUS RESEARCH INSTITUTE ENTEBBE
RESEARCH ETHICS COMMITTEE
APPROVED
22 FEB 2022
P. O. BOX 49, ENTEBBE
UGANDA
