# Supplementary material for: Understanding the social and physical menstrual health environment of secondary schools in Uganda: A qualitative methods study
Source: PLOS Glob Public Health. 2023 Nov 29;3(11):e0002665. doi: 10.1371/journal.pgph.0002665 (PMC10686490; doi:10.1371/journal.pgph.0002665)
Supplement: S6 Text — (PDF) [file pgph.0002665.s006.pdf]

Annex 5A. Draft Semi - structured Interview Guide for female students for the MENISCUS Rapid Assessment study

Date: **Start time (24hr format):** **End time:**

School code:

Facilitator's names

Primary objectives:

1) To gain a deeper understanding of perspectives of the social and physical environment relevant to menstrual health
2) To explore the functioning of existing students' committees and groups at the school.

**Topic Guide Themes for interview**
1. Background information about the informant<br>2. Description of the school environment in relation to menstruation and illness management<br>3. What activities and groups or committees do students take part in?<br>4. How are WASH facilities maintained at the school?<br>5. Perceptions of the interventions, support structures and facilities related to WASH and reproductive or menstrual health in the school or in the wider community<br>6. Any further issues raised in the group discussions with students<br><br>*Note: Topics could change or be revised following observations carried out during the rapid assessment.*

**Closing Questions**
1. Do you have anything else would like to add to the discussion that we have not yet covered?
2. Do you have any questions for me? I may not be able to answer them all, but I will do my best.
**To end our discussion, I want to thank you for your time and speaking with me today. I really learned a lot from you!**<br><br>**Before we leave today, is there something you would like to talk about? Is there something we talked about today that you would like to know more about?**<br><br>**Thank you for participating.**

UGANDA VIRUS RESEARCH INSTITUTE ENTEBBE
RESEARCH ETHICS COMMITTEE
APPROVED
22 FEB 2022
P. O. BOX 49, ENTEBBE
UGANDA
