# Supplementary material for: Understanding the social and physical menstrual health environment of secondary schools in Uganda: A qualitative methods study
Source: PLOS Glob Public Health. 2023 Nov 29;3(11):e0002665. doi: 10.1371/journal.pgph.0002665 (PMC10686490; doi:10.1371/journal.pgph.0002665)
Supplement: S7 Text — (PDF) [file pgph.0002665.s007.pdf]

Annex 5C. Draft Semi-Structured Interview Guide for senior teachers for the MENISCUS Rapid Assessment study

Date: **Start time (24hr format):** **End time:**

School code:

Facilitator's names:

Primary objectives:

1) To gain a deeper understanding of perspectives of the social and physical environment relevant to menstrual health
2) To explore functioning of existing parents, students, teachers' committee at the school.

**Topic Guide Themes for interview**
1. Background information about the informant.<br>2. Description of the school environment in relation to menstruation and illness management.<br>3. What groups or committees exist in the school and what are their roles? How are WASH facilities maintained at the school?<br>4. Perceptions of the interventions, support structures and facilities related to WASH and reproductive or menstrual health in the school or in the wider community.<br>5. Recommendations for introducing and delivering new activities and programmes in the school.<br>6. Explore any further issues raised in the group discussions or interviews with students.<br><br>*Note: Topics could change or be revised following observations carried out during the rapid assessment.*

**Closing Questions**
1. Do you have anything else would like to add to the discussion that we have not yet covered?
2. Do you have any questions for me? I may not be able to answer them all, but I will do our best.
**To end our discussion, I want to thank you for your time and speaking with me today. I really learned a lot from you!**<br><br>**Before we leave today, is there something you would like to talk about? Is there something we talked about today that you would like to know more about?**<br><br>**Thank you for participating.**

UGANDA VIRUS RESEARCH INSTITUTE ENTEBBE
RESEARCH ETHICS COMMITTEE
APPROVED
22 FEB 2022
P. O. BOX 49, ENTEBBE
UGANDA
