# Supplementary material for: Understanding the social and physical menstrual health environment of secondary schools in Uganda: A qualitative methods study
Source: PLOS Glob Public Health. 2023 Nov 29;3(11):e0002665. doi: 10.1371/journal.pgph.0002665 (PMC10686490; doi:10.1371/journal.pgph.0002665)
Supplement: S8 Text — (PDF) [file pgph.0002665.s008.pdf]

Telephone Interview Guide female student V1.0 June2021

The Rapid assessment of secondary school physical and social environments for menstrual health in Uganda

1. Introduction and consenting:

Call the participant on the phone number available on file

Introduce yourself (name and MRC/UVRI & LSHTM Uganda Research Unit)

Check if you are speaking to the participant and ask how she is. If the parent/caregiver answers the phone explain to them the purpose of your call first, and if they agree, ask to speak to their child.

Ask if this is a good time to call or if the participant would prefer you to call her back at a different day and time (if so, note time and date and end the call explaining you will call back at the agreed time). Ask if the participant is comfortable where she is at the time of call, or if she would like to move to another room or move outside.

If the participant agrees to speak on phone at the time of your call, explain the participant that you are calling to ask a few questions about your perceptions of the school social and physical environment relevant to menstrual health and explore the functioning of existing students' committees and groups at your school.

Explain to the participant that she is free to choose if she wants to answer the questions, and that her parent has already consented to her participation in the study.

If the participant agrees, take verbal assent from the participant and proceed to the interview.

Start asking the following questions;

2. Interview questions:

i. Background information about the informant
ii. Description of the school environment in relation to menstruation and illness management
iii. What activities and groups or committees do students take part in?
iv. How are WASH facilities maintained at the school?
v. Are there separate toilets for boys and girls? What kind of toilets are there?
vi. Is water usually available at school. If so, what kind of water source(s)?
vii. Perceptions of the interventions, support structures and facilities related to WASH and reproductive or menstrual health in the school or in the wider community
viii. Any further issues raised in the group discussions with students

3. Closing questions:

i. Do you have anything else would like to add to the discussion that we have not yet covered?
ii. Do you have any questions for me? I may not be able to answer them all, but I will do my best.

UVRI-RESEARCH ETHICS COMMITTEE
VALID UNTIL
22 FEB 2022
APPROVED
P. O. BOX 49, ENTEBBE (U)

iii. Is there something you would like to talk about? Is there something we talked about today that you would like to know more about?

4. Ending the telephone call

Thank the participant for participating in the phone interview and ask if they have any other questions.

Explain to the participant that you will phone her again in case regular study activities cannot yet resume due to the COVID19 outbreak and related response.

End the phone call with 'stay safe, stay home'.

UVRI-RESEARCH ETHICS COMMITTEE
VALID UNTIL
22 FEB 2022
APPROVED
P. O. BOX 49, ENTEBBE (U)
