# Supplementary material for: Understanding the social and physical menstrual health environment of secondary schools in Uganda: A qualitative methods study
Source: PLOS Glob Public Health. 2023 Nov 29;3(11):e0002665. doi: 10.1371/journal.pgph.0002665 (PMC10686490; doi:10.1371/journal.pgph.0002665)
Supplement: S10 Text — (PDF) [file pgph.0002665.s010.pdf]

Telephone Interview Guide senior teachers V1.0 June2021

The Rapid assessment of secondary school physical and social environments for menstrual health in Uganda

Date: **Start time (24hr format):** **End time:**

School code:

Facilitator's names:

1. **Introduction and consenting:**

Call the participant on the phone number available on file

Introduce yourself (name, job title, MRC/UVRI & LSHTM Uganda Research Unit)

Check if you are speaking to the participant and ask how he/she is.

Ask if this is a good time to call or if the participant would prefer you to call him/her back at a different day and time (if so, note time and date and end the call explaining you will call back at the agreed time). Ask if the participant is comfortable where he/she is at the time of call, or if he/she would like to move to another room or move outside.

If the participant agrees to speak on phone at the time of your call, explain the participant that you are calling to ask about a few questions on your perceptions of the school social and physical environment relevant to menstrual health and explore the functioning of existing parents, students and teachers' committee at the school.

Explain to the participant that he/she is free to choose if he / she wants to answer the questions.

If the participant agrees, take verbal consent from the participant and proceed to the interview.

Start asking the following questions.

2. **Interview questions:**
   - i. Background information about the informant.
   - ii. Description of the school environment in relation to menstruation and illness management.
   - iii. What groups or committees exist in the school and what are their roles? How are WASH facilities maintained at the school?
   - iv. Perceptions of the interventions, support structures and facilities related to WASH and reproductive or menstrual health in the school or in the wider community.
   - v. Recommendations for introducing and delivering new activities and programmes in the school.
   - vi. Explore any further issues raised in the group discussions or interviews with students

UVRI-RESEARCH ETHICS COMMITTEE
VALID UNTIL
22 FEB 2022
APPROVED
P. O. BOX 49, ENTEBBE (U)

3. Closing questions:

i. Do you have anything else would like to add to the discussion that we have not yet covered?
ii. Do you have any questions for me? I may not be able to answer them all, but I will do my best.
iii. Is there something you would like to talk about? Is there something we talked about today that you would like to know more about?

4. Ending the telephone call

Thank the participant for participating in the phone interview and ask if they have any other questions.

Explain the participant that you will phone him/her again in case regular study activities cannot yet resume due to the COVID19 outbreak and related response.

End the phone call with 'stay safe, stay home'.

UVRI-RESEARCH ETHICS COMMITTEE
VALID UNTIL
22 FEB 2022
APPROVED
P. O. BOX 49, ENTEBBE (U)
